# Mouse B cells engineered to express an anti-HPV antibody elicit anti-tumor T cell responses

**DOI:** 10.3389/fimmu.2025.1613879

**Published:** 2025-07-18

**Authors:** Michal Guberman Bracha, Guy Biber, Natalie Zelikson, Sharon Shavit, Roy Avraham, Yaron Vagima, Débora Rosa Bublik, Yael Katz, Adi Barzel, Leah Natasha Klapper, Shmuel Hess, Alessio David Nahmad

**Affiliations:** ^1^ Tabby Therapeutics Ltd, Ness Ziona, Israel; ^2^ Faculty of Life Sciences, Tel Aviv University, Tel Aviv, Israel; ^3^ Faculty of Medical & Health Sciences, Tel Aviv University, Tel Aviv, Israel; ^4^ Department of Biotechnology, Israel Institute for Biological Research, Ness Ziona, Israel; ^5^ The Samueli Integrative Cancer Pioneering Institute, Petah Tikva, Israel

**Keywords:** B cell, antibody, cancer, cell therapy (CT), tertiary lymphoid structures, genome editing, cell engineering

## Abstract

Transplantation of engineered B cells has demonstrated efficacy in HIV disease models. B cell engineering may also be utilized for the treatment of cancer. Recent studies have highlighted that B cell activity is associated with favorable clinical outcomes in oncology. In mice, polyclonal B cells have been shown to elicit anti-cancer responses. As a potential novel cell therapy, we demonstrate that engineering B cells to target a tumor-associated antigen enhances polyclonal anti-tumor responses. We observe that engineered B cells expressing an anti-HPV B cell receptor internalize the antigen, enabling subsequent activation of oncoantigen-specific T cells. Secreted antibodies from engineered B cells form immune complexes, which are taken up by antigen-presenting cells to further promote T cell activation. Engineered B cells hold promise as novel, multi-modal cell therapies and open new avenues in solid tumor targeting.

## Introduction

Recent progress in prophylactic vaccination has reduced Human Papilloma Virus (HPV) infection rates (1). However, a rise in the incidence of HPV-associated head and neck cancers has been recorded (2, 3). Novel therapeutic vaccines are being developed but are not designed for long-term persistence or localized responses (4, 5). In addition, increased vaccine hesitancy reduces population-wide prophylactic effects, even in light of improved vaccine safety (6). T Cell Receptor (TCR) engineered T cells targeting the HPV E6 (7) or E7 (8) oncoantigens have been tested in the clinic but are limited by human leukocyte antigen (HLA) matching and have shown escape following major histocompatibility complex (MHC) mutations (9, 10). These challenges may be addressed through B cell engineering.

Multiple *ex vivo* B cell engineering approaches have recently been developed (11–17), demonstrating efficacy as emerging therapeutics in viral disease models (18–23). B cell modalities may not require conventional lymphodepletion prior to transfer, thereby reducing the burden of care (24). *In vivo* B cell or hematopoietic stem cell editing further broadens the therapeutic potential of B cell engineering strategies (25, 26). Nuclease mediated IgH engineering in B cells enables the hijacking of endogenous constant exons via appropriate splicing signals and allows well-regulated expression of the transgenic antibody. The transgene is first expressed as a membrane-bound B cell receptor (BCR) and subsequently, following differentiation into progeny plasmablasts and plasma cells, as a secreted, soluble antibody. IgH targeting also enables memory retention, class-switch recombination, somatic hypermutation, and clonal selection (13, 18, 22, 23). Beyond viral infections, tertiary lymphoid structures (TLS)s (27–30) and B cell-associated responses (31–34) are linked to favorable prognosis and promote responses to immunotherapy in cancer (35–37). In particular, B cell signatures are associated with favorable responses in HPV-positive head and neck cancers (38–40). Polyclonal, antigen-specific, or tumor-activated B cells have been shown to induce T cell responses (41, 42), potentiate cytotoxic activity and reduce tumor burden in mice (43–45). Finally, enhancing immunogenicity and polyclonal T cell responses against tumors may benefit patients, as indicated by clinical trials combining checkpoint inhibitors and chemotherapy (37, 46).

Therefore, we chose to engineer B cells to target HPV E6. E6 is a viral antigen; thus, targeting it should limit off-tumor activity. We also hypothesized that, because E6 is a tumorigenic antigen, it would be less likely to be downregulated as an escape mechanism (47). Finally, compared to other dominant HPV oncoantigen E7, E6 is a longer protein and may therefore enable presentation of more epitopes for T cell activation, potentially leading to more potent immunogenic cascades.

Herein, we describe a B cell engineering approach to target the HPV-associated oncoantigen E6. We demonstrate, for the first time, that mouse engineered B cells (EBC) activate polyclonal E6-reactive T cells, potentially promoting anti-tumor responses.

## Results

### Engineering strategy and mode of action

**Figure 1:**
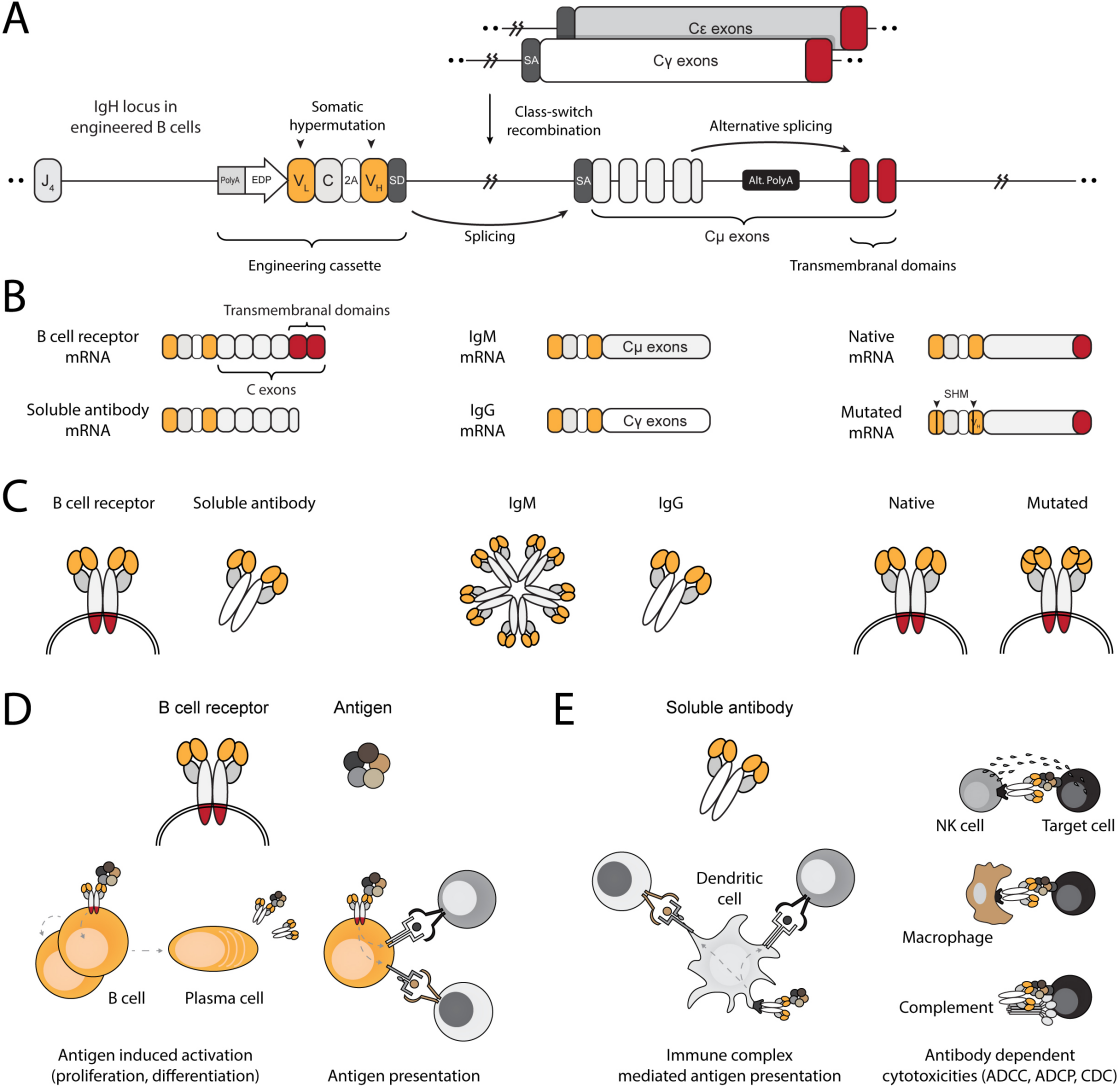
Engineering B cells at the IgH locus to enable multi-modal adaptive immune responses. **(A)** Schematic of the engineered IgH locus in EBCs. The cassette is integrated downstream of the final J segment and upstream of the constant segments. To downregulate endogenous expression, a PolyA signal is added to the 5’ end the cassette, followed by an enhancer dependent promoter (EDP) to upregulate expression upon on-target integration. The coding segment includes a full light chain (V_L_–C) separated by a 2A peptide from a variable fragment of the heavy chain (V_H_). A splice donor (SD) at the 3’ end of the cassette enables splicing with the splice acceptor (SA) of the endogenous constant segments, regardless of the isotype expressed in the EBC. **(B)** mRNA-level expression of the engineering cassette in EBCs. Since it does not include constant segments, the resulting therapeutic antibody expressed in EBCs may be in the form of a BCR when alternative splicing enables incorporation of the membranal exons, or as a soluble antibody when the alternative polyA (Alt. PolyA) is activated in antibody-secreting cells (left). Splicing with endogenous constant segments further enables EBCs to express the antibody as virtually any available isotype (middle). Finally, since the engineering cassette is introduced into the native IgH gene, it can undergo somatic hypermutation (SHM), potentially improving antigen-binding affinity through clonal selection (right). **(C)** Same as **(B)**, but represented at the protein level. **(D)** Schematic representation of antibody functions when expressed on the membrane as a BCR. Effector functions of the BCR in EBCs enable antigen-induced activation, leading to EBC proliferation and differentiation into antibody-secreting cells or antigen-presenting cells. The BCR internalizes antigens for processing into peptides, which are then loaded onto MHC complexes. These are subsequently presented to T cells, enabling mutually beneficial interactions. **(E)** Schematic representation of the antibody after secretion. The soluble antibody binds to soluble antigens, forming immune complexes that facilitate T cell activation via Fc receptor-expressing cells, such as dendritic cells (left). The soluble antibody can bind to membrane-expressed antigens, enabling a range of cytotoxic reactions such as antibody-dependent cell cytotoxicity (ADCC), antibody-dependent cell phagocytosis (ADCP), and complement-dependent cytotoxicity (CDC).

We engineer the IgH locus of B cells using electroporation of RNA-guided nucleases and transduction with recombinant adeno-associated viral vectors (rAAV). The bicistronic cassette encodes an anti-HPV E6 full light chain and the variable domain of the heavy chain. The translated segments are separated by a 2A peptide and terminated with a splice donor. Successful integration into the J–C intron of the IgH locus allows expression and splicing with endogenous constant segments, leading to translation of a full heavy chain (**Figure 1A**). Therefore, EBCs express the anti-HPV E6 antibody as a BCR and can differentiate into plasmablasts and plasma cells, secreting the anti-HPV E6 antibody (**Figures 1B, C**). As a BCR, the antibody facilitates B cell activation and internalization of the bound antigen for processing and presentation on MHC, ultimately leading to antigen-specific T cell activation (**Figure 1D**). When secreted by differentiated EBCs, the antibody further enables antigen-specific T cell activation through the formation of immune complexes (**Figure 1E**). Moreover, since the edited target locus is located upstream of the genomic regions that undergo rearrangement during class-switch recombination, the anti-HPV E6 antibody can undergo isotype switching. Isotype switching enables mucosal tissue protection, systemic clearance, immune complex-mediated T cell activation, and effector functions such as antibody-dependent cellular cytotoxicity (48). Finally, because the cassette is integrated within the IgH locus, it may undergo somatic hypermutation during germinal center (GC) reactions, enabling clonal expansion and potentially leading to affinity maturation.

### Robust mouse B cell engineering

**Figure 2:**
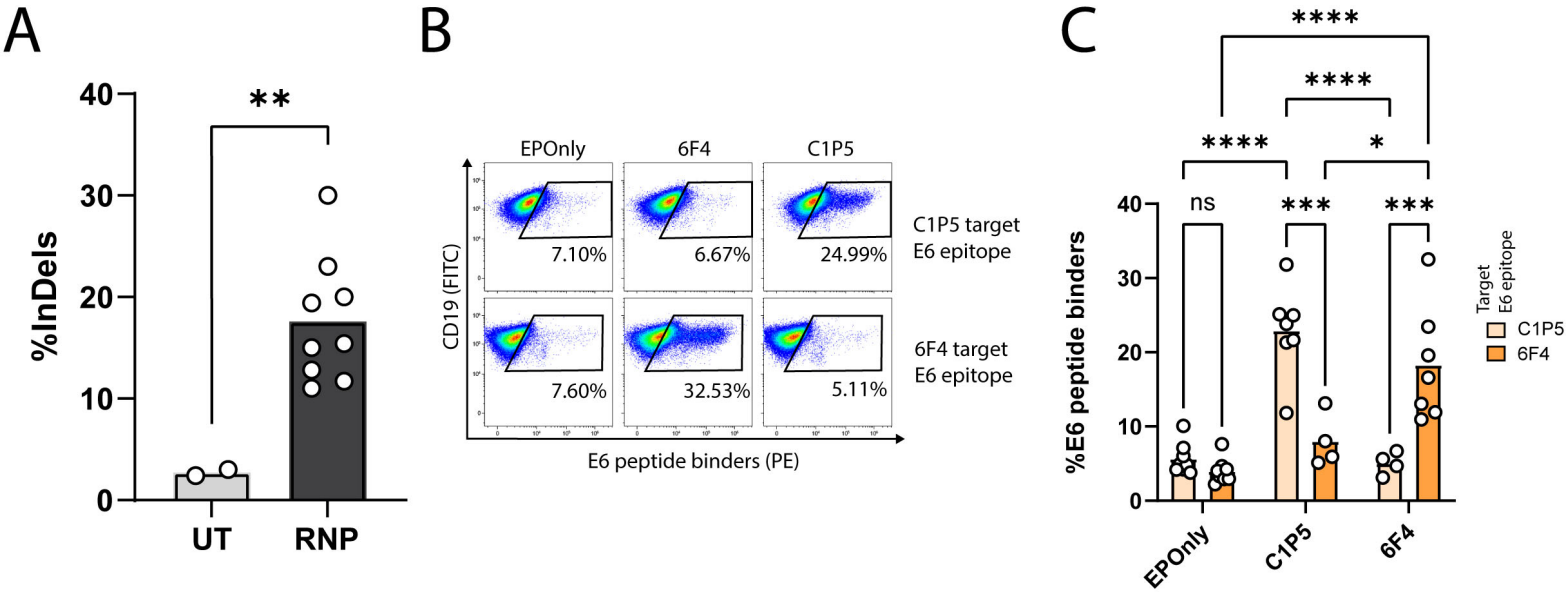
Engineering primary mouse B cells to target HPV-E6. **(A)** TIDE analysis of mouse splenic lymphocytes edited at the IgH locus. CRISPR-Cas9 activity is detected by insertions and deletions (InDels). Cultured lymphocytes (UT) are compared to cells electroporated with CRISPR-Cas9 ribonucleoproteins (RNPs). Each dot represents an independent experiment, *n* = 2–9. **pv<0.01, unpaired two-tailed *t*-test. **(B)** Representative flow cytometry of mouse splenic lymphocytes engineered to express either the 6F4 (middle column) or C1P5 (right column) anti-HPV-E6 antibodies, compared to cells electroporated but not transduced with AAVs (EPOnly, left column). Engineering rates were assessed using peptide containing either the C1P5 target epitope in the E6 protein (top row) or the 6F4 target epitope (bottom row), both derived from the E6 protein. Pre-gated on live, singlet, CD19^+^ cells. **(C)** Quantification of **(B)**, showing engineering rates of primary mouse splenic lymphocytes expressing either the C1P5 or 6F4 anti-HPV-E6 antibodies compared to control EPOnly B cells. To assess specificity, cells were stained using either the C1P5 target epitope (Navajo white) or the 6F4 target epitope (orange), both derived from the E6 protein. Each dot represents an independent experiment, *n* = 4–8. ns pv>0.05, *pv<0.05, ***pv<0.001, ****pv<0.0001, two-way ANOVA with Tukey’s multiple comparisons test.

We engineered B cells to express the anti-E6 antibodies C1P5 or 6F4 (49, 50). CRISPR/Cas9 activity, enabling on-target double-strand breaks for downstream integration of the engineering cassette, was confirmed using TIDE (**Figure 2A**). Since our engineering cassette does not encode the constant domains of the heavy chain, including membrane-anchoring domains, on-target genome integration enables BCR expression via splicing with endogenous heavy chain segments (18, 21, 22). We therefore quantified functional engineering rates by spectral cytometry using E6 peptides containing the target epitope for either C1P5 or 6F4 (**Figures 2B, C**).

We further characterized subsets of the engineered cells by spectral cytometry. No significant phenotypic differences between EBCs and non-engineered B cells were detected. Three main B cell populations were identified. The dominant population exhibited an unswitched, activated B cell phenotype with GC homing potential, comprising up to 63% of the cells. In addition, antibody-secreting cells and class-switched B cells, made up 11% and 3% of the cell populations, respectively (**Supplementary Figure 1**). Employing i.29 cells, which basally express low levels of the kappa light chain, enabled detection of engineered cells independently of antigen binding. When engineered to express 6F4, the cells could reliably bind the full E6 protein, but not when engineered to express C1P5 (**Supplementary Figures 2A, B**). 6F4 bound the E6 protein at lower concentrations than C1P5 (**Supplementary Figure 2C**). The E6 transcript undergoes alternative splicing to a shorter version called E6*I. This splicing event is thought to support E7 expression (51). Interestingly, the 6F4 binding site—but not the C1P5 binding site—is present in both the full-length E6 and the alternatively spliced E6*I protein product (**Supplementary Figure 2D**).

Therefore, we proceeded with the 6F4 binder and improved the primary B cell engineering protocol by enriching B cells prior to culture, employing standardized culture media and electroporating the cells at a lower concentration (**Supplementary Figures 2E, F**). Taken together, these results confirm that IgH targeting is a robust approach for engineering B cells to target tumor-associated antigens; however, expression level, antibody affinity, binding epitope, and target expression must be considered when selecting a therapeutically relevant antibody for B cell engineering.

### EBCs act as APCs to elicit antigen-specific T cell responses

**Figure 3:**
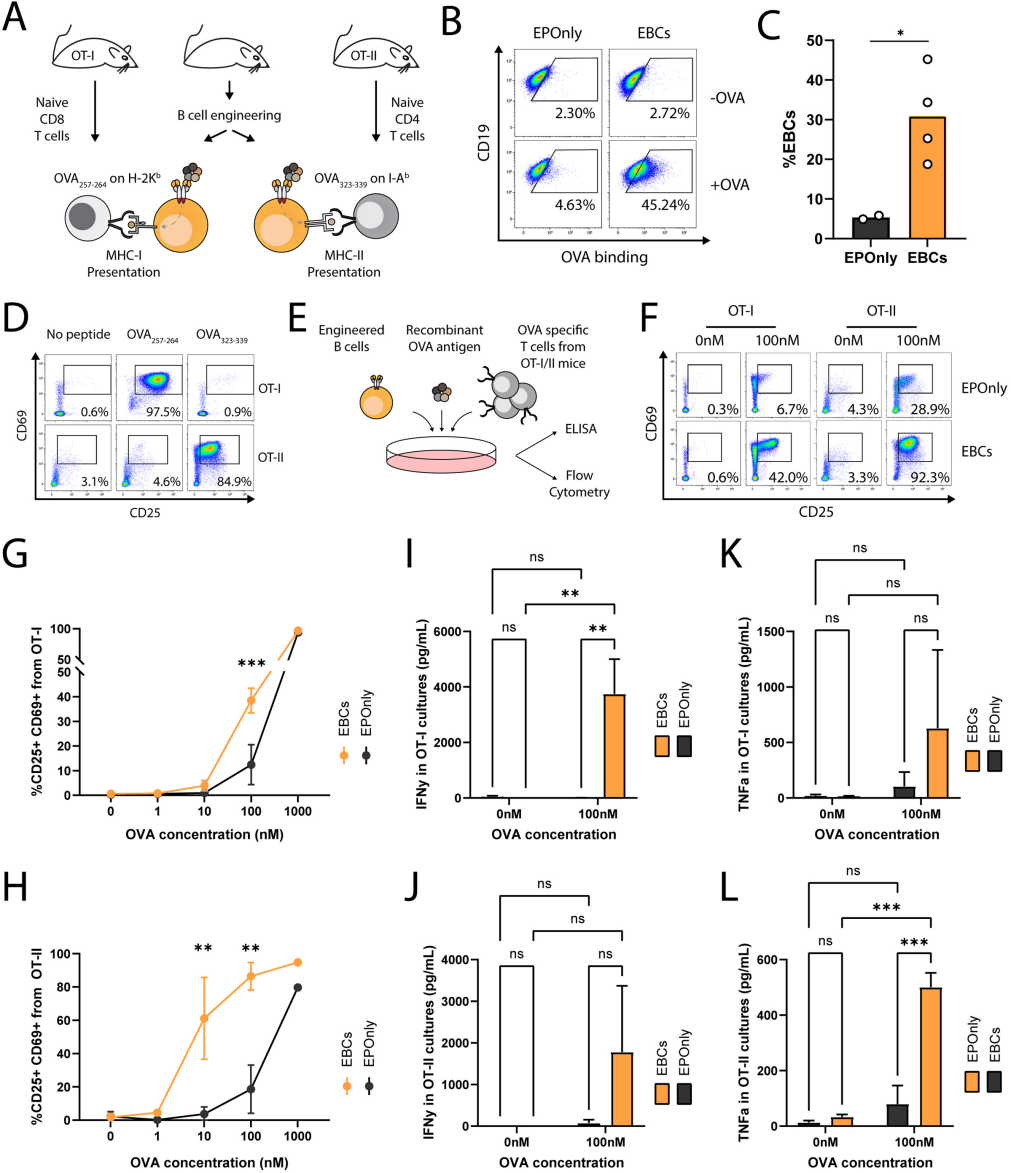
EBCs activate CD4 and CD8 T cell responses **(A)** Schematic representation of the ovalbumin system used in these experiments. B cells were extracted from immunocompetent mice for the production of OVA-specific EBCs. T cells were isolated from OT-I or OT-II mice, which are transgenic for Class I- or Class II-restricted TCRs specific to OVA, respectively. **(B)** Representative flow cytometry of OVA-specific engineering. EBCs were compared to cells electroporated with RNPs but not transduced with AAVs (EPOnly). Cells were then with or without the OVA antigen. Binding was detected only in electroporated and transduced cells in the presence of the OVA antigen. Pre-gated on live, singlet, CD19^+^ cells. **(C)** Quantification of **(B)** for the +OVA samples. Each dot represents an independent experiment, *n* = 2–4. *pv<0.05, unpaired two-tailed *t*-test. **(D)** Representative flow cytometry of OT-I and OT-II T cells extracted from mice and incubated with APCs pulsed with either the OVA_257–264_ or the OVA_323–339_ peptides, recognized by OT-I and OT-II T cells, respectively, but not reciprocally. Pre-gated on singlets, live cells, CD19^−^, CD4^+^ CD8^−^ (for OT-II co-cultures) and CD8^+^ CD4^−^ (for OT-I co-cultures). **(E)** Schematic of the co-culture setup. Engineered B cells were loaded with the OVA antigen and co-cultured with either OT-I or OT-II T cells. **(F)** Representative flow cytometry of activation markers CD25 and CD69 in OT-I (left) and OT-II (right) co-cultures with EPOnly control cells (top) or EBCs (bottom), pre-loaded with either 0 nM or 100 nM of the OVA antigen. Pre-gated on singlets, live cells, CD4^+^ CD8^−^ (for OT-II co-cultures) and CD8^+^ CD4- (for OT-I co-cultures). Numbers in the plots indicate the percentage of CD69^+^ CD25^+^ cells. **(G)** Quantification of **(F)** for OT-I co-cultures, including data with concentrations of OVA ranging from 0 nM to 1,000 nM. **(H)** Quantification of **(F)** for OT-II co-cultures, including data with concentrations of OVA ranging from 0 nM to 1,000 nM. For **(G, H)**, bars represent the mean, error bars indicate SD, *n* = 2–4, pooled data from two independent experiments. **pv<0.01, ***pv<0.001, two-way ANOVA with Tukey’s multiple comparisons test. **(I)** IFNg concentrations in the supernatants of OT-I co-cultures. **(J)** IFNg concentrations in the supernatants of OT-II co-cultures. **(K)** TFNa concentrations in the supernatants of OT-I co-cultures. **(L)** TFNa concentrations in the supernatants of OT-II co-cultures. For **(I–L)**, bars represent the mean, error bars indicate SD, *n* = 2–4, pooled from two independent experiments. ns pv >0.05, **pv<0.01, ***pv<0.001, two-way ANOVA with uncorrected Fisher’s LSD.

B cells are potent antigen-presenting cells (APCs) (52). Interaction between B cells and T cells require HLA compatibility and TCR specificity (53). Previous studies have shown that polyclonal and antigen-specific B cells can activate CD4^+^ and CD8^+^ T cells (42–44, 54). However, mouse models with T cells recognizing E6 via a defined TCR are lacking. To initially demonstrate antigen-induced activation of T cells by mouse EBCs *in vitro*, we engineered B cells to express the OB-I antibody, a specific binder for ovalbumin (OVA) (55), and employed the OT-I and OT-II systems as cognate CD8^+^ and CD4^+^ T cells, respectively (**Figure 3A**) (56, 57). We achieved approximately 30% engineering efficiency, as detected by flow cytometry (**Figures 3B, C**). OT-I T cells recognize Ova_257–264_ presented on MHC class I H-2K^b^, and OT-II T cells recognize Ova_323–339_ presented on MHC class II I-A^b^. Thus, we validated each T cell type by co-culturing with peptide-spiked, non-engineered primary mouse B cells (**Figure 3D**). Next, we co-cultured OB-I EBCs, with the full OVA antigen and either OT-I or OT-II T cells (**Figures 3A, E**). B cells are professional APCs and may therefore present antigens independently of antibody expression, likely via pinocytosis (58). However, antigen presentation to be significantly more efficient for B cells expressing an antibody specific to the antigen (59). Thus, we investigated dose responses to full OVA antigen pre-incubated with EBCs prior to co-culture, as monitored by T cell activation markers and compared to control non-engineered B cells. As detected by flow cytometry and ELISA, significantly higher activation of OT-I and OT-II cells was observed when using OB-I EBCs compared to control non-engineered B cells (**Figures 3F–L**). Indeed, we detected increased frequencies of T cells expressing activation markers CD69 and CD25, along with elevated secretion of IFNg and TNFa in the supernatants of EBC-T cell co-cultures supplemented with OVA antigen. This effect was particularly evident at antigen concentrations of 10 nM–100 nM, indicating that the antibody expressed in EBCs internalizes OVA for processing and presentation on MHC class I and II. At high antigen concentration, pinocytosis may enable B cells to uptake-up antigens for T cell presentation, independent of antibody specificity (44, 59). Consistently, we observed activation of OT-I and OT-II T cells in co-cultures with EPOnly B cells at 1,000 nM OVA. Together, these results demonstrate that at low antigen concentrations, EBCs function as potent antigen-specific-presenting cells.

### EBCs elicit polyclonal, antigen-specific T cell responses

**Figure 4:**
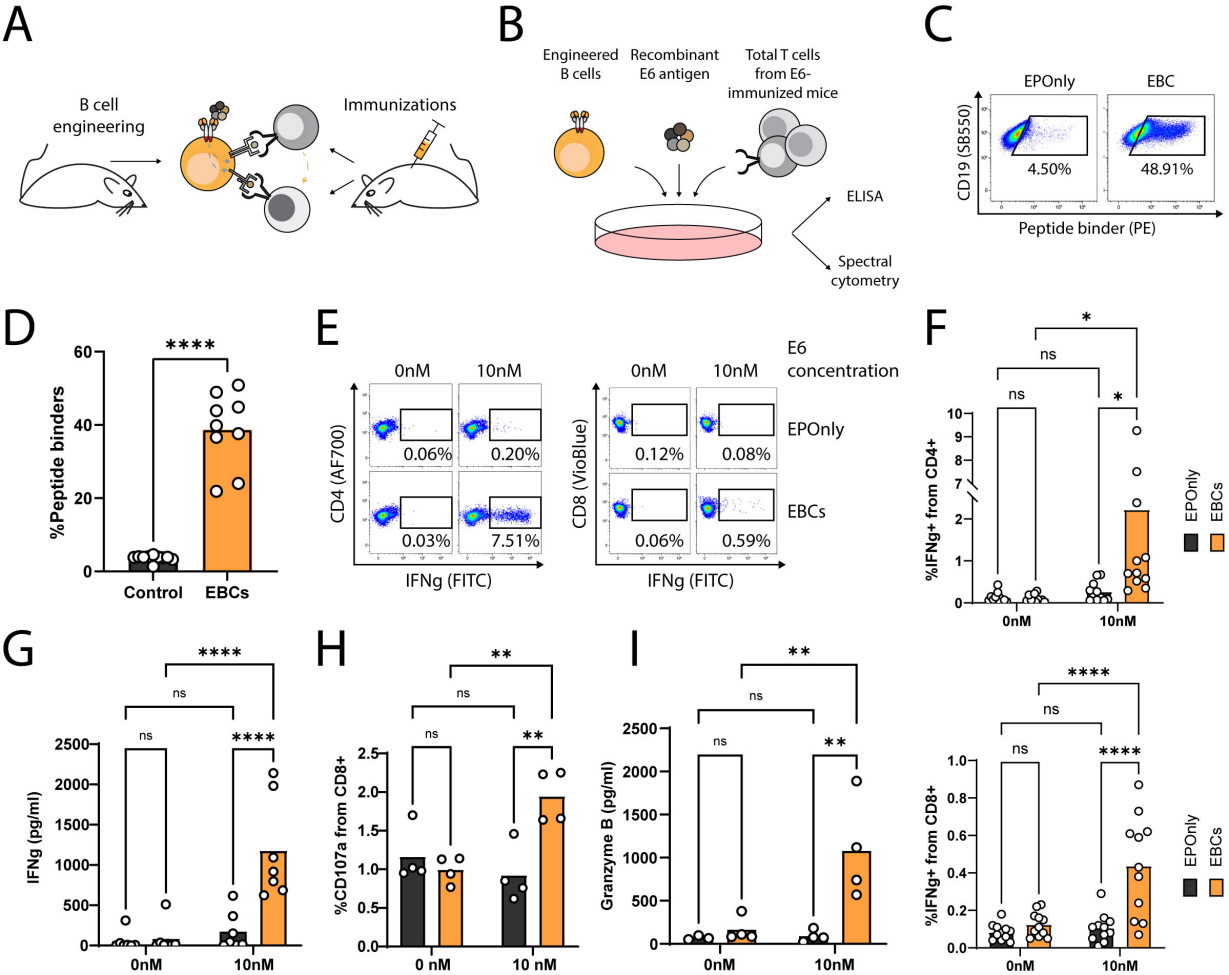
EBCs elicit polyclonal anti-tumor T cell responses. **(A)** Schematic representation of the E6 co-cultures experiments. Mice were immunized three times to generate polyclonal anti-E6 T cells. Independent mice were used as a source for B cell engineering. B cells internalize antigens for presentation to CD4^+^ and CD8^+^ T cells (gray arrow). In co-cultures containing CD4^+^ and CD8^+^ T cells along with B cells, CD8^+^ T cell activation may be further enhanced by cytokine-mediated help from CD4^+^ T cells (orange arrow). **(B)** Experimental scheme of the co-cultures. EBCs or EPOnly control cells were pre-incubated with the E6 antigen and then added to total T cells from immunized mice. **(C)** Representative flow cytometry of EBCs engineered to target E6. EBCs were detected with an E6 peptide containing the target epitope of the 6F4 binder. Pre-gated on live, singlet, CD19^+^ cells. **(D)** Quantification of **(C)**. Each dot represents an independent experiment, *n* = 9–10. ****pv<0.0001, unpaired two-tailed *t*-test. **(E)** Representative intracellular flow cytometry for IFNg in CD4^+^ (left) or CD8^+^ (right) cells from polyclonal co-cultures with EPOnly control cells (above) or EBCs (below) at either 0 nM or 10 nM E6. Pre-gated on singlets, cells, alive, CD4^+^ CD8^−^ (left) or CD8^+^ CD4^−^ (right). **(F)** Quantification of **(E)** for CD4^+^ (top) or CD8^+^ (bottom) T cells. Each dot represents an independent co-culture using either EBC (orange) or EPOnly control cells (black), *n* = 11. Pooled data from five independent experiments. ns pv >0.05, *pv<0.05, ****pv<0.0001, two-way ANOVA with Šidák’s multiple comparisons tests. **(G)** ELISA of IFNg secretion from supernatants of T cells from immunized mice co-cultured with EPOnly control B cells (black) or E6-specific EBCs (orange). ns pv >0.05, ****pv<0.0001, two-way ANOVA with Šidák’s multiple comparisons tests. Each dot represents an independent co-culture; data pooled from three individual experiments, *n* = 7. **(H)** Degranulation of cytotoxic T cells in co-cultures [as in **(B)**], monitored by CD107a. Pre-gated on singlets, live cells, CD4^−^ CD8^+^. ns pv >0.05, **pv<0.01, two-way ANOVA with Šidák’s multiple comparisons tests. Each dot represents an independent co-culture, *n* = 4. **(I)** ELISA of Granzyme B in cells from immunized mice co-cultured with either EPOnly control B cells (black) or E6-specific EBCs (orange) as in **(B)** at 0 nM or 10 nM antigen. ns pv >0.05, **pv<0.01, two-way ANOVA with Tukey’s multiple comparison. Each dot represents an independent co-culture, *n* = 4.

To generate polyclonal, E6-reactive T cells, we immunized mice with the E6 oncoantigen and extracted total T cells following three immunizations (**Figures 4A, B**). To demonstrate the APC functions of mouse EBCs *in vitro*, we co-incubated E6-immunized splenic T cells with engineered splenic B cells (**Figures 4C, D**) in the presence of the full E6 oncoantigen. Following internalization, EBCs present peptides derived from the proteolysis of the oncoantigen on MHC class I/II, subsequently activating E6-reactive T cells. As indicated by intracellular flow cytometry on CD4^+^ and CD8^+^ T cells (**Figures 4E, F**; **Supplementary Figure 3A**) and confirmed by ELISA (**Figure 4G**) for IFN-γ expression and secretion, EBCs—but not control non-engineered B cells (EPOnly)—activate T cells in the presence of E6 at concentrations as low as 10 nM. Furthermore, EBCs enhanced CD8^+^ cytotoxicity, as indicated by granzyme B and upregulation of the degranulation marker CD107a (**Figures 4H, I**; **Supplementary Figure 3B**). We compared T cell activation elicited by mouse EBCs and control EPOnly B cells when cultured at E6 concentrations ranging from 0.1 nM to 100 nM. These experiments indicated that at high E6 concentrations, EPOnly B cells could also activate E6-reactive T cells, possibly via non-specific, low-efficiency pinocytosis. Importantly, EBCs initiated T cell activation at concentrations 10–100 times lower than those required for EPOnly control B cells (**Supplementary Figures 3A–C**).

### EBCs secrete antibodies further inducing T cell activation

**Figure 5:**
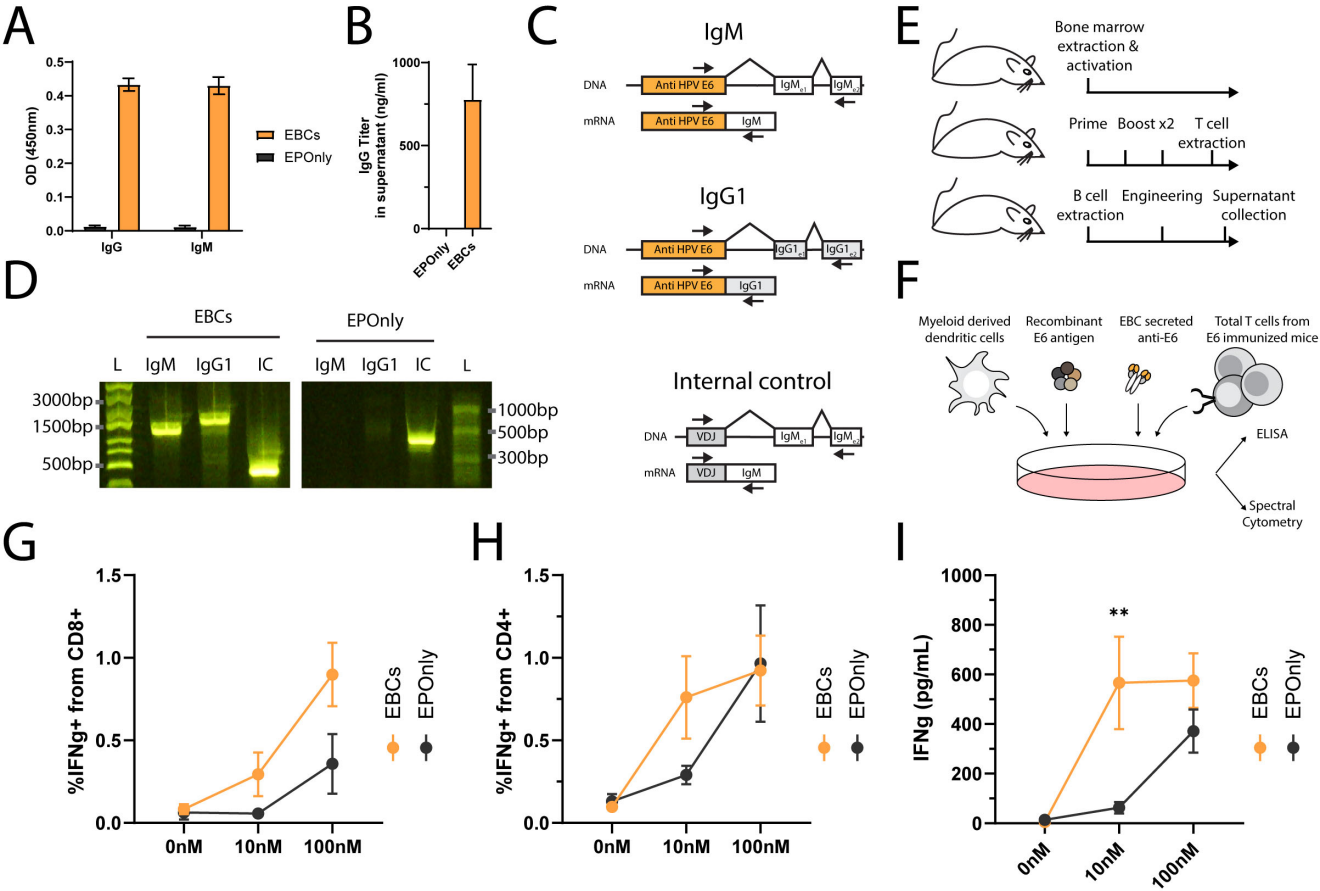
Immune complexes derived from antibodies secreted by EBCs activate T cells. **(A)** Isotype-specific ELISA for antibodies against E6 in the supernatants of B cells engineered as in **Figure 2A**. Mean OD values and SD are shown for IgG (left) or IgM (right) E6-specific antibodies from EBC (orange) or EPOnly (black) cells, *n* = 2–3. **(B)** Quantification of IgG data from **(A)** using a recombinant IgG1 antibody. Mean and SD values are represented. Pooled data from two independent experiments, *n* = 2. **(C)** Representative PCR on reverse-transcribed mRNA from EBCs. Primer binding sites are indicated with arrows. The anti-E6 engineering cassette are shown in orange, and endogenous segments in white or gray. For each amplicon, the DNA (top) or mRNA (bottom) are illustrated. Only spliced fragments derived from mRNA are amplified due to polymerization length constraints. **(D)** Gel electrophoresis of amplicons from **(C)**, using RNA extracted from EBCS (left) or mRNA from EPOnly control B cells (right). Molecular weight ladders with relative sizes are shown beside each gel. **(E)** Representative schematic of cell sources used in **(F–I)**. Bone marrow from naïve mice was used to generate myeloid-derived dendritic cells. T cells were obtained from mice immunized with E6. B cells for engineering were isolated from separate naïve mice. **(F)** Representative experimental scheme for **(G–I)**. Myeloid-derived dendritic cells were seeded and loaded with immune complexes formed by incubating E6 antigen with EBC-derived supernatant. T cells from E6-immunized mice were then added to the culture. **(G)** Intracellular flow cytometry for IFNg in CD8^+^ T cells from cultures as in **(F)**, incubated with supernatants from either EBCs (orange) or EPOnly control B cells (black). Pre-gated on singlets, live cells, CD4^−^ CD8^+^. **(H)** Same as **(G)**, but for CD4^+^ CD8^−^ T cells. **(I)** ELISA for IFNg in supernatants from cocultures as described in **(F)**. For **(G–I)**, mean and SEM are represented, *n* = 5–7, pooled from three independent experiments. **pv<0.01 for two-way ANOVA with Šidák’s multiple comparisons tests.

In general, secreted antibodies may form immune complexes that enable T cell activation. The antibody binds to the soluble antigen, and the resulting immune complexes are internalized by Fc receptor-expressing APCs, enabling cross-presentation (48, 60, 61). Mouse EBCs secrete polyisotypic anti-HPV E6 antibodies, as detected by ELISA (**Figures 5A, B**) and RT-PCR, further confirming molecular evidence of on-target integration (**Figures 5C, D**). To simulate the immune complex process *in vitro*, we incubated EBC supernatants with the antigen and loaded them onto myeloid-derived dendritic cells. Splenic T cells from immunized mice were subsequently added to the dendritic cell cultures and monitored for activation by ELISA (**Figures 5E, F**). EBC supernatants formed immune complexes that enabled T cell activation (**Figures 5G–I**).

## Discussion

Eliciting an adaptive, tumor-specific, and polyclonal response has recently emerged as a challenge in cell therapy. This challenge arose from the low response rates of monoclonal therapeutics targeting solid tumors and was underlined by studies employing additional cargo immune effector cargos, such as cytokines that activate endogenous responses (62, 63). In GCs and TLSs, B cell and T cell interactions are mutually beneficial and are associated with favorable prognosis in many solid tumors (35, 36, 52). Therefore, exploiting, enhancing, or producing TLSs presents promising opportunities in cancer immunotherapy.

B cell engineering provides a platform to trigger and enhance multi-modal anti-tumor activity. Multiple academic and industry groups (24, 64) are working on EBC applications for viral infections, protein therapy, and cancer (13, 15, 17, 18, 21, 23, 65). Here, we uniquely demonstrate that B cells engineered to express an anti-HPV antibody targeting an intracellular oncoantigen can potentiate T cell responses. When expressing a BCR, the engineered B cells uptake antigens, enabling the activation of CD8 and CD4 T cells. We demonstrated this effect using both monoclonal antigen-specific T cells and polyclonal T cells from immunized mice. When differentiated into antibody-secreting cells, engineered B cells produce antibodies that form immune complexes with the targeted antigen. These immune complexes subsequently facilitate antigen-specific T cell activation.

These results highlight that engineered B cell activity is best assessed in conjunction with other immune cell interactions. Thus, the therapeutic potential of our approach may need to be assessed in rare mouse models that support TLS formation (31), or in larger animal models (64). An independent study demonstrates that human engineered B cells may activate anti-HPV T cells and that targeting a membrane-bound antigen may enable additional effector functions such as ADCC, CDC or ADCP (66). Efficacy may be further enhanced from encoding an additional payload in the integrated cassette. Additionally, previous reports have indicated that antibody mispairing may occur in conventionally engineered B cells. Therefore, future modifications may include encoding the antibody as a single chain to reduce mispairing with the endogenous light chain, as previously reported (13, 21, 22). Such single-chain encoding may also enable the expression of a bispecific antibody targeting two different oncoantigens Expression of the HPV-E6 antigen in mouse models (67, 68) and human tumors (69) is low to undetectable. Therefore, from a translation perspective, bispecific B cell engineering may improve sensitivity and enable activation of a broader T cell repertoire. AAV-free engineering (70), recombinase-mediated engineering (71), or nuclease-free engineering (72) may reduce production costs. *In vivo* B cell engineering may further simplify processes, shorten production time, and enable an off-the-shelf alternative (26). Finally, B cell engineering as a platform technology may be applied in the future to treat diverse conditions, including congenital diseases and autoimmune disorders.

## Materials and methods

### Construct design

Constructs were designed as previously characterized, specifically for integration into the IgH gene following a nuclease-mediated double-strand break (22, 26). Donor cassettes encode an antibody expressed under the Enhancer Dependent Promoter (EDP, mutated IGH promoter) (22), with a splice donor to the endogenous heavy chain, thus allowing expression only upon on-target integration into the IgH locus. The segment is preceded by a bGH PolyA signal to ablate endogenous VDJ expression. The variable domains of the antibody are preceded by IgK and IgH leaders. The leaders were pre-spliced to avoid intronic coding and splicing interference. The light chain employs the IgK constant and is separated from the heavy chain using a Furin-GSG-P2A sequence, allowing ribosomal skipping and efficient protein separation. The variable region of the heavy chain is followed by the intronic portion of the native IgHJ4 splice donor, enabling splicing with the endogenous heavy chain exons. The sequences were verified to ensure frame conservation throughout the fully expressed RNA, including at the splice junction with the endogenous constant segments. The coding sequences of the constructs were codon-optimized using a custom pipeline to enhance expression in mice cells, ablate cryptic splice sites, remove G-quadruplets, and improve somatic hypermutation hotspots (22). All donor constructs were delivered using single- stranded AAV backbones and packaged into AAV-DJ serotypes (Packgene Biotech). **Supplementary Table 1** lists the sequences, origins, and components of an example construct.

### B cell engineering

For initial studies involving the binders presented in **Figure 2**, B cells were engineered as previously described (22). For E6 co-culture experiments, mouse splenic B cells from naïve 5–9-week-old C57BL/6 mice were negatively selected with B cell isolation kit (Miltenyi Biotec) seeded in Immunocult mouse B cell expansion kit (Stem Cell Technologies) and incubated at 37°C and 5% CO_2_ for 1 day. Cells were subsequently electroporated using the NEON Transfection System (ThermoFisher Scientific). Each electroporation contained 100 pmol sgRNA and 30.5 pmol Alt-R HiFi CRISPR-Cas9 (IDT) in Buffer R, using settings 1,675 V, 10 ms, three pulses for 0.5e6 cells per 10 uL reaction. AAV-DJ containing custom cassettes (PackGene Biotech) was added to the cells within 5 min post-electroporation at an MOI of 100,000. Following electroporation, cells were incubated for 24 h, then supplemented with 1 mL of Immunocult Mouse B Cell Expansion Kit media and incubated for an additional 24 h before downstream experiments and analyses.

A list of reagents used for cell culture and engineering is provided in **Supplementary Tables 2**, **3**.

### Mice

All mouse experiments complied with ethical regulations under the supervision of the Ministry of Health, Israel. OT-I and OT-II mice were obtained from Charles River Laboratories. Spleen and bone marrow from C56BL/6 mice were obtained from Science in Action Ltd. To generate polyclonal anti-E6 T cells, 6–12-week-old mice (Envigo) were immunized intraperitoneally with recombinant E6 (Abcam) every 2–4 weeks at a dose of 20 ug/200 uL/mouse in 1% Alhydrogel (Invivogen) for a total of three immunizations. Mice were housed at ambient temperatures of 19 ˚C–23 ˚C, 45%–65% humidity, and a 12 h light/12 h dark cycle.

### Flow cytometry

To detect engineering via E6 target epitopes of the respective binders, cells were incubated at 0.5 μM in Cell Staining Buffer (Biolegend) with biotinylated peptides or biotinylated recombinant E6 protein (R&D Systems) after staining for Zombie L/D (Biolegend), washed and stained with streptavidin (Biolegend) and other antibodies for phenotypic characterization. For OB-I engineering, cells were incubated with OVA at 1 mg/mL in Cell Staining Buffer for 10 min at room temperature following Zombie staining, washed, incubated with rabbit polyclonal anti-OVA (1:50 dilution), washed again, and detected using conjugated anti-rabbit (1:50 dilution). In general, L/D staining was performed according to the manufacturer’s instructions (Biolegend), and mouse-specific conjugated antibodies were used at a 1:100 dilution in Cell Staining Buffer (Biolegend) for 10 min at room temperature. Samples were acquired using a Cytek Northern Lights V/B/R spectral cytometer (Cytek Biosciences). For flow cytometry gating of engineered cells, the biological control—commonly EPOnly—was used to set the gate at values<10%. Cells from the EBC samples within that gate were considered engineered. Positive cells in EPOnly controls may result from technical, nonspecific binding, or from naïve polyclonal B cells expressing diverse BCRs and SMVT/SLC5A6, which can bind biotin. Biotinylation of recombinant E6 was performed according to the manufacturer’s instructions (ThermoFisher). N-terminal biotinylated peptides were synthesized by GenScript.

A list of antibodies and reagents used in detection experiments is provided in **Supplementary Table 2**. Custom peptide sequences are provided in **Supplementary Table 3**.

### Phenotype analyses

For unbiased spectral analysis of cell phenotypes, computational analysis was performed using the *Spectre* R package (73). Samples were initially prepared in FlowJo, and data were exported as raw-value CSV files. The datasets were merged into a single data table, with sample origin (EBCs and EPOnly) annotated, following exclusion of Zombie Yellow^+^ and CD45.1^−^ cells. The *FlowSOM* algorithm (74) was then applied to the merged dataset to cluster the data into four cell populations. Clustering was based on the expression of the following markers: CD19, CD138, CD27, CD80, CD86, IgD, IgM, and IgG. Cluster annotation was performed manually. Subsequently, data were analyzed using the *Uniform Manifold Approximation and Projection (UMAP)* algorithm (75) for cellular visualization. The expression distribution of each marker within each cluster was analyzed and exported as csv files for bar plot visualization. Plots were generated using the *Spectre::make.colour.plot* and *ggplot2* functions (76).

For analysis of E6 binding from engineered cells populations, quantification was performed as follows:


$$\%E6\;binders\;from\;engineered\;cells=100\;\times \;\frac{\left(Fraction\;of\;IgK^{+}E6^{binding}\right)}{\left(Fraction\;of\;IgK^{+}E6^{non-binding}\right)+\left(Fraction\;of\;IgK^{+}E6^{binding}\right)}$$

### Antigen presentation assays

Antigen presentation assays of co-cultures were performed at a 1:1 ratio in 96-well plates for 48 h. Cells were seeded in cell culture medium at a 1:1 ratio, with a final total cell concentration of 1e6 cells/mL. For APC experiments using EBCs, B cells 2–3 days post-engineering were pre-incubated with the indicated concentration of E6 (R&D systems) for 20 min at 37 ˚C and 5%CO_2_ prior to T cell supplementation. For co-cultures with polyclonal or OT-I/II derived T cells, splenic lymphocytes were negatively enriched for T cells (Miltenyi) prior to seeding onto B cells.

For immune complex experiments, mouse bone marrow cells were lysed to remove red blood cells (Biolegend) and cultured with 20 ngmL GM-CSF (peprotech) and 10 ng/mL IL-4 (Miltenyi) in RPMI 1640 supplemented with 10% FBS (SigmaAldrich), 2 nM L-glutamine (Sartorius), 100 u/mL penicillin, 100 ug/ml streptomycin, and 50 µM β-mercaptoethanol (SigmaAldrich). Suspended cells were carefully removed on day 3, and adherent cells were cultured for an additional 3 days. Immune complexes were formed by incubating E6 (R&D Systems) with antibodies derived from the supernatants of EPOnly or EBCs, collected 2 days post-engineering, at the indicated concentrations for 20 min at 37 ˚C. Immune complexes were then directly loaded onto counted and reseeded myeloid-derived dendritic cells, followed by the addition of T cells as described above.

A list of reagents employed is provided in **Supplementary Table 2**.

### ELISA

ELISA for IFNg, TNFa, GrzmB on the supernatant of co-cultures were performed according to the kit manufacturer’s instructions (R&D Systems). For isotype specific ELISA, plates were coated with E6 (R&D Systems) at 2ug/ml in PBS for 24h at 4˚C. Blocking was performed in 5% BSA, for 2h at room temperature. Supernatant dilutions ranged 1:2-1:20 depending on the specific sample. Secondary HRP conjugated anti-mouse IgG or anti-mouse IgM (Jackson Immunoresearch) were utilized at a 1:5000 dilution and development was acquired on a Multiskan FC Microplate photometer (ThermoScientific). ELISA for EBC secreted anti-HPV-E6 antibodies were quantified using a commercially available recombinant version of the antibody (Abcam) serially diluted to produce a standard curve.

### Nucleic acid manipulations

For TIDE analysis of on-target double-strand breaks, gDNA was extracted using the DNeasy Blood and Tissue Kit (Qiagen). PCR was performed for 35 cycles using PrimeStar MAX (Takara). Primers sequences are listed in **Supplementary Table 3**. Resulting amplicons were purified using AMPure XP beads (Beckman Coulter) at a bead-to-sample 1:1 ratio. Purified amplicons were subjected to Sanger sequencing at Hylabs Ltd. Samples were analyzed using the TIDE algorithm (77).

For reverse translated PCR reactions of cassette integration and expression, total RNA was extracted using the RNeasy Mini Kit (Qiagen) with on-column DNAse treatment. Reverse transcription was performed using RevertAid (ThermoFisher) and oligodT primers. PCR on the resulting complementary DNA was performed for 35 cycles using PrimeStar MAX DNA Polymerase (Takara). Following each PCR, resulting amplicons were analyzed agarose gel electrophoresis using a standard DNA ladder, and imaged with the E-Gel Power Snap Electrophoresis System (ThermoFisher).

### Statistical analysis and illustration

Statistical analyses and data visualization were performed on distinct biological samples using GraphPad Prism version 10. Two-tailed unpaired t-tests were performed for comparisons between two groups. For multiple group comparisons, p-values were adjusted for multiplicity using appropriate correction. Following ANOVA, Tukey’s, Šídák’s, or Dunnett’s *post hoc* tests were applied based on the comparisons indicated in each figure. Each figure legend specifies the statistical test used, the measure of tendency, and the type of error bars. Final visualizations were created using Adobe Illustrator version 29.4.

## Data Availability

The raw data supporting the conclusions of this article will be made available by the authors, without undue reservation.

## References

[1] Joura EA Giuliano AR Iversen OE Bouchard C Mao C Mehlsen J. A 9-valent HPV vaccine against infection and intraepithelial neoplasia in women. N Engl J Med. (2015) 372(8):711–23. doi: 10.1056/NEJMoa1405044, PMID: 25693011

[2] Marur S D’Souza G Westra WH Forastiere AA. HPV-associated head and neck cancer: A virus-related cancer epidemic. Lancet Oncol. (2010) 11(8):781–9. doi: 10.1016/S1470-2045(10)70017-6, PMID: 20451455 PMC5242182

[3] Näsman A Du J Dalianis T. A global epidemic increase of an HPV-induced tonsil and tongue base cancer – potential benefit from a pan-gender use of HPV vaccine. J Intern Med. (2020) 287(2):134–52., PMID: 31733108 10.1111/joim.13010

[4] Blagovic K Smith CK Ramakrishnan A Moore L Soto DR Thompson Z. Engineered red blood cells (activating antigen carriers) drive potent T cell responses and tumor regression in mice. Front Immunol. (2022) 13:1–21. doi: 10.3389/fimmu.2022.1015585, PMID: 36263022 PMC9573954

[5] Ramos da Silva J Bitencourt Rodrigues K Formoso Pelegrin G Silva Sales N Muramatsu H de Oliveira Silva M. Single immunizations of self-amplifying or non-replicating mRNA-LNP vaccines control HPV-associated tumors in mice. Sci Transl Med. (2023) 15(686):eabn3464. doi: 10.1126/scitranslmed.abn3464, PMID: 36867683

[6] Sonawane K Lin YY Damgacioglu H Zhu Y Fernandez ME Montealegre JR. Trends in human papillomavirus vaccine safety concerns and adverse event reporting in the United States. JAMA Netw Open. (2021) 4(9):1–13. doi: 10.1001/jamanetworkopen.2021.24502, PMID: 34533574 PMC8449282

[7] Draper LM Kwong MLM Gros A Stevanović S Tran E Kerkar S. Targeting of HPV-16+ epithelial cancer cells by TCR gene engineered T cells directed against E6. Clin Cancer Res. (2015) 21(19):4431–9. doi: 10.1158/1078-0432.CCR-14-3341, PMID: 26429982 PMC4603283

[8] Jin BY Campbell TE Draper LM Stevanović S Weissbrich B Yu Z. Engineered T cells targeting E7 mediate regression of human papillomavirus cancers in a murine model. JCI Insight. (2018) 3(8). doi: 10.1172/jci.insight.99488, PMID: 29669936 PMC5931134

[9] Doran SL Stevanović S Adhikary S Gartner JJ Jia L Kwong MLM. T-cell receptor gene therapy for human papillomavirus-associated epithelial cancers: A first-in-human, phase I/II study. J Clin Oncol. (2019) 37(30):2759–68. doi: 10.1200/JCO.18.02424, PMID: 31408414 PMC6800280

[10] Nagarsheth NB Norberg SM Sinkoe AL Adhikary S Meyer TJ Lack JB. TCR-engineered T cells targeting E7 for patients with metastatic HPV-associated epithelial cancers. Nat Med. (2021) 27(3):419–25. doi: 10.1038/s41591-020-01225-1, PMID: 33558725 PMC9620481

[11] Fusil F Calattini S Amirache F Mancip J Costa C Robbins JB. A lentiviral vector allowing physiologically regulated membrane-anchored and secreted antibody expression depending on B-cell maturation status. Mol Ther. (2015) 23(11):1734–47. doi: 10.1038/mt.2015.148, PMID: 26281898 PMC4817946

[12] Ou T He W Quinlan BD Guo Y Karunadharma P Park H. Reprogramming of the heavy-chain CDR3 regions of a human antibody repertoire. Mol Ther. (2021) 30(1):184–97. doi: 10.1016/j.ymthe.2021.10.027, PMID: 34740791 PMC8753427

[13] Rogers GL Huang C Mathur A Huang X Chen H. Reprogramming human B cells with custom heavy chain antibodies. Nat Biomed Eng. (2024), 1700–14. doi: 10.1038/s41551-024-01240-4, PMID: 39039240

[14] Voss JE Gonzalez-Martin A Andrabi R Fuller RP Murrell B McCoy LE. Reprogramming the antigen specificity of B cells using genome-editing technologies. Elife. (2019) 8(e42995). doi: 10.7554/eLife.42995, PMID: 30648968 PMC6355199

[15] Page A Delles M Nègre D Costa C Fusil F Cosset F. Engineering B cells with customized therapeutic responses using a synthetic circuit. Mol Ther - Nucleic Acids. (2023) 33:1–14. doi: 10.1016/j.omtn.2023.05.024, PMID: 37359346 PMC10285500

[16] Hung KL Meitlis I Hale M Chen CY Singh S Jackson SW. Engineering protein-secreting plasma cells by homology-directed repair in primary human B cells. Mol Ther. (2018) 26(2):456–67. doi: 10.1016/j.ymthe.2017.11.012, PMID: 29273498 PMC5835153

[17] Ueda N Cahen M Leonard J Deleurme L Dreano S Sirac C. Single-hit genome editing optimized for maturation in B cells redirects their specificity toward tumor antigens. Sci Rep. (2024) 14(1):1–12. doi: 10.1038/s41598-024-74005-3, PMID: 39342013 PMC11438885

[18] Huang D Tran JT Olson A Vollbrecht T Guryleva MV Tenuta M. Vaccine elicitation of HIV broadly neutralizing antibodies from engineered B cells. Nat Commun. (2020), 1–10. doi: 10.1038/s41467-020-19650-8, PMID: 33203876 PMC7673113

[19] Rogers GL Cannon PM. Genome edited B cells: a new frontier in immune cell therapies. Mol Ther. (2021) 29(11):3192–204. doi: 10.1016/j.ymthe.2021.09.019, PMID: 34563675 PMC8571172

[20] Hartweger H McGuire AT Horning M Taylor JJ Dosenovic P Yost D. HIV-specific humoral immune responses by CRISPR/Cas9-edited B cells. J Exp Med. (2019) 216(6):1301–10. doi: 10.1084/jem.20190287, PMID: 30975893 PMC6547862

[21] Moffett HF Harms CK Fitzpatrick KS Tooley MR Boonyaratanakornkit J Taylor JJ. B cells engineered to express pathogen-specific antibodies protect against infection. Sci Immunol. (2019) 4(35). doi: 10.1126/sciimmunol.aax0644, PMID: 31101673 PMC6913193

[22] Nahmad AD Raviv Y Horovitz-fried M Sofer I Akriv T Nataf D. Engineered B cells expressing an anti-HIV antibody enable memory retention, isotype switching and clonal expansion. Nat Commun. (2020), 1–10. doi: 10.1038/s41467-020-19649-1, PMID: 33203857 PMC7673991

[23] Yin Y Yan G Jiang Y Quinlan B Peng H Crynen G. *In vivo* affinity maturation of murine B cells reprogrammed to express human antibodies. Nat Biomed Eng. (2024). doi: 10.1038/s41551-024-01179-6, PMID: 38486104 PMC12090756

[24] Sheridan C. B cells as drug factories. Nat Biotechnol. (2024). doi: 10.1038/s41587-024-02283-3, PMID: 38886605

[25] Li C Anderson AK Wang H Gil S Kim J Huang L. Stable HIV decoy receptor expression after *in vivo* HSC transduction in mice and NHPs: Safety and efficacy in protection from SHIV. Mol Ther. (2023) 31(4):1059–73. doi: 10.1016/j.ymthe.2023.02.002, PMID: 36760126 PMC10124088

[26] Nahmad AD Lazzarotto CR Zelikson N Kustin T Tenuta M Huang D. *In vivo* engineered B cells secrete high titers of broadly neutralizing anti-HIV antibodies in mice. Nat Biotechnol. (2022) 40:1241–1249. doi: 10.1038/s41587-022-01328-9, PMID: 35681059 PMC7613293

[27] Meylan M Petiprez F Becht E Bougouin A Pupier G Calvez A. Tertiary lymphoid structures generate and propagate anti-tumor antibody-producing plasma cells in renal cell cancer. Immunity. (2022) 55. doi: 10.1016/j.immuni.2022.02.001, PMID: 35231421

[28] Helmink BA Reddy SM Gao J Zhang S Basar R Thakur R. B cells and tertiary lymphoid structures promote immunotherapy response. Nature. (2020) 577(7791):549–55. doi: 10.1038/s41586-019-1922-8, PMID: 31942075 PMC8762581

[29] Cabrita R Lauss M Sanna A Donia M Skaarup Larsen M Mitra S. Tertiary lymphoid structures improve immunotherapy and survival in melanoma. Nature. (2020) 577:561–5. doi: 10.1038/s41586-019-1914-8, PMID: 31942071

[30] Vanhersecke L Brunet M Guégan JP Rey C Bougouin A Cousin S. Mature tertiary lymphoid structures predict immune checkpoint inhibitor efficacy in solid tumors independently of PD-L1 expression. Nat Cancer. (2021) 2(8):794–802. doi: 10.1038/s43018-021-00232-6, PMID: 35118423 PMC8809887

[31] Cui C Wang J Chen min P Connolly K Damo M Fagerberg E. Neoantigen driven B cell and CD4+ T follicular helper cell collaboration promotes robust anti-tumor CD8+ T cell responses. Cell. (2021) 184:1–18. doi: 10.1016/j.cell.2021.11.007, PMID: 34852236 PMC8671355

[32] Ng KW Boumelha J Enfield KSS Almagro J Cha H Pich O. Antibodies against endogenous retroviruses promote lung cancer immunotherapy. Nature. (2023) 616:563–573. doi: 10.1038/s41586-023-05771-9, PMID: 37046094 PMC10115647

[33] Petitprez F de Reynies A Keung EZ Chen T Sun C Calderaro J. B cells are associated to sarcoma survival and immunotherapy response. Nature. (2020) 577:556–60. doi: 10.1038/s41586-019-1906-8, PMID: 31942077

[34] Germain C Gnjatic S Tamzalit F Knockaert S Remark R Goc J. Presence of B cells in tertiary lymphoid structures is associated with a protective immunity in patients with lung cancer. Am J Respir Crit Care Med. (2014) 189(7):832–44. doi: 10.1164/rccm.201309-1611OC, PMID: 24484236

[35] Sautès-Fridman C Petitprez F Calderaro J Fridman WH. Tertiary lymphoid structures in the era of cancer immunotherapy. Nat Rev Cancer. (2019) 19:307–25. doi: 10.1038/s41568-019-0144-6, PMID: 31092904

[36] Fridman WH Meylan M Petitprez F Sun CM Italiano A Sautès-Fridman C. B cells and tertiary lymphoid structures as determinants of tumour immune contexture and clinical outcome. Nat Rev Clin Oncol. (2022) 0123456789. doi: 10.1038/s41571-022-00619-z, PMID: 35365796

[37] Italiano A Bessede A Pulido M Bompas E Piperno-Neumann S Chevreau C. Pembrolizumab in soft-tissue sarcomas with tertiary lymphoid structures: a phase 2 PEMBROSARC trial cohort. Nat Med. (2022) 28(6):1199–206. doi: 10.1038/s41591-022-01821-3, PMID: 35618839

[38] Ruffin AT Cillo AR Tabib T Liu A Onkar S Kunning SR. B cell signatures and tertiary lymphoid structures contribute to outcome in head and neck squamous cell carcinoma. Nat Commun. (2021) 12. doi: 10.1038/s41467-021-23355-x, PMID: 34099645 PMC8184766

[39] Ruffin AT Li H Vujanovic L Zandberg DP Ferris RL Bruno TC. Improving head and neck cancer therapies by immunomodulation of the tumour microenvironment. Nat Rev Cancer. (2022) 23:173–188. doi: 10.1038/s41568-022-00531-9, PMID: 36456755 PMC9992112

[40] Wieland A Patel MR Cardenas MA Eberhardt CS Hudson WH Obeng RC. Defining HPV-specific B cell responses in patients with head and neck cancer. Nature. (2020) 597:274–278. doi: 10.1038/s41586-020-2931-3, PMID: 33208941 PMC9462833

[41] Bruno TC Ebner PJ Moore BL Squalls OG Waugh KA Eruslanov EB. Antigen-presenting intratumoral B cells affect CD4+ TIL phenotypes in non–small cell lung cancer patients. Cancer Immunol Res. (2017) 5(10):898–907. doi: 10.1158/2326-6066.CIR-17-0075, PMID: 28848053 PMC5788174

[42] de Wit J Souwer Y Jorritsma T Bos HK Brinke ten A Neefjes J. Antigen-specific B cells reactivate an effective cytotoxic T Cell response against phagocytosed Salmonella through cross-presentation. PloS One. (2010) 5(9):2–11. doi: 10.1371/journal.pone.0013016, PMID: 20885961 PMC2946406

[43] Lee-Chang C Miska J Hou D Rashidi A Zhang P Burga RA. Activation of 4-1BBL+ B cells with CD40 agonism and IFNγ elicits potent immunity against glioblastoma. J Exp Med. (2020) 218., PMID: 32991668 10.1084/jem.20200913 PMC7527974

[44] Wennhold K Thelen M Schlößer HA Haustein N Reuter S Garcia-Marquez M. Using antigen-specific B cells to combine antibody and T cell–based cancer immunotherapy. Cancer Immunol Res. (2017) 5:730–43. doi: 10.1158/2326-6066.CIR-16-0236, PMID: 28778961

[45] Li Q Lao X Pan Q Ning N Yet J Xu Y. Adoptive transfer of tumor reactive B cells confers host T-cell immunity and tumor regression. Clin Cancer Res. (2011) 17:4987–95. doi: 10.1158/1078-0432.CCR-11-0207, PMID: 21690573 PMC3149727

[46] Harrington KJ Burtness B Greil R Soulières D Tahara M De Castro G. Pembrolizumab with or without chemotherapy in recurrent or metastatic head and neck squamous cell carcinoma: updated results of the phase III KEYNOTE-048 study. J Clin Oncol. (2023) 41:790–802. doi: 10.1200/JCO.21.02508, PMID: 36219809 PMC9902012

[47] Hoppe-Seyler K Bossler F Braun JA Herrmann AL Hoppe-Seyler F. The HPV E6/E7 oncogenes: key factors for viral carcinogenesis and therapeutic targets. Trends Microbiol. (2018) 26:158–68. doi: 10.1016/j.tim.2017.07.007, PMID: 28823569

[48] Wang XY Wang B Wen YM. From therapeutic antibodies to immune complex vaccines. NPJ Vaccines. (2019) 4:1–8. doi: 10.1038/s41541-018-0095-z, PMID: 30675393 PMC6336872

[49] Jiang Z Albanese J Kesterson J Warrick J Karabakhtsian R Dadachova E. Monoclonal antibodies against human papillomavirus E6 and E7 oncoproteins inhibit tumor growth in experimental cervical cancer. Transl Oncol. (2019) 12(10):1289–95. doi: 10.1016/j.tranon.2019.06.003, PMID: 31325765 PMC6642219

[50] Giovane C Trave G Briones A Lutz Y Wasylyk B Weiss E. Targetting of the N-terminal domain of the human papillomavirus type 16 E6 oncoprotein with monomeric ScFvs blocks the E6-mediated degradation of cellular p53. J Mol Recognit. (1999). doi: 10.1002/(SICI)1099-1352(199903/04)12:2<141::AID-JMR453>3.0.CO;2-O, PMID: 10398405

[51] Tang S Tao M McCoy JP Zheng Z-M. The E7 oncoprotein is translated from spliced E6*I transcripts in high-risk human papillomavirus type 16- or type 18-positive cervical cancer cell lines via translation reinitiation. J Virol. (2006) 80:4249–63. doi: 10.1128/JVI.80.9.4249-4263.2006, PMID: 16611884 PMC1472016

[52] Fridman WH Petitprez F Meylan M Chen TWW Sun CM Roumenina LT. B cells and cancer: To B or not to B? J Exp Med. (2021) 218:1–11. 10.1084/jem.20200851 PMC7754675 33601413

[53] Hong S Zhang Z Liu H Tian M Zhu X Zhang Z. B cells are the dominant antigen-presenting cells that activate naive CD4+ T cells upon immunization with a virus-derived nanoparticle antigen. Immunity. (2018) 49(4):695–708.e4. doi: 10.1016/j.immuni.2018.08.012, PMID: 30291027

[54] Hou D Katz JL Lee-Chang C. Generation of B-cell-based cellular vaccine for cancer in murine models. STAR Protoc. (2023) 4(2):102219. doi: 10.1016/j.xpro.2023.102219, PMID: 37083319 PMC10322876

[55] Avalos AM Bilate AM Witte MD Tai AK He J Frushicheva MP. Monovalent engagement of the BCR activates ovalbumin-specific transnuclear B cells. J Exp Med. (2014) 211:365–79. doi: 10.1084/jem.20131603, PMID: 24493799 PMC3920557

[56] Hogquist KA Jameson SC Heath WR Howard JL Bevan MJ Carbone FR. T cell receptor antagonist peptides induce positive selection. Cell. (1994) 76:17–27. doi: 10.1016/0092-8674(94)90169-4, PMID: 8287475

[57] Barnden MK Allison J Heath WR Carbone FR. Defective TCR expression in transgenic mice constructed using cDNA-based a- and b-chain genes under the control of heterologous regulatory elements. Immunol Cell Biol. (1998) 76:34–40. doi: 10.1046/j.1440-1711.1998.00709.x, PMID: 9553774

[58] Avalos AM Ploegh HL. Early BCR events and antigen capture, processing, and loading on MHC class II on B cells. Front Immunol. (2014) 5:1–5. doi: 10.3389/fimmu.2014.00092, PMID: 24653721 PMC3948085

[59] Singer DF Linderman JJ. The relationship between antigen concentration, antigen internalization, and antigenic complexes: Modeling insights into antigen processing and presentation. J Cell Biol. (1990) 111:55–68. doi: 10.1083/jcb.111.1.55, PMID: 2365735 PMC2116156

[60] Inoue T Shinnakasu R Kawai C Yamamoto H Sakakibara S Ono C. Antibody feedback contributes to facilitating the development of Omicron-reactive memory B cells in SARS-CoV-2 mRNA vaccinees. J Exp Med. (2023) 220(2):1–15. doi: 10.1084/jem.20221786, PMID: 36512034 PMC9750191

[61] Hägglöf T Cipolla M Loewe M Chen ST Mesin L Hartweger H. Continuous germinal center invasion contributes to the diversity of the immune response. Cell. (2022), 147–61. doi: 10.1016/j.cell.2022.11.032, PMID: 36565698 PMC9825658

[62] Pegram HJ, Lee JC, Hayman EG, Imperato GH, Tedder TF, Sadelain M. Tumor-targeted T cells modified to secrete IL-12 eradicate systemic tumors without need for prior conditioning. Blood. (2012) 119:4133–41. doi: 10.1182/blood-2011-12-400044, PMID: 22354001 PMC3359735

[63] Arnett AB Fleurence J Sweidan R Wang T Zhang H Thakkar SG. Interleukin-15-armoured GPC3 CAR T cells for patients with solid cancers. Nature. (2024). doi: 10.1038/s41586-024-08261-8, PMID: 39604730 PMC12704925

[64] Young DJ Edwards AJ Quiroz Caceda KG Liberzon E Barrientos J Hong S. *In vivo* tracking of ex vivo generated 89 Zr-oxine labeled plasma cells by PET in a non-human primate model. Mol Ther. (2024) 33:580–94., PMID: 39741408 10.1016/j.ymthe.2024.12.042 PMC11852699

[65] Cheng hong Hung RY KL Zhang T Stoffers CM Ott AR Suchland ER. Ex vivo engineered human plasma cells exhibit robust protein secretion and long-term engraftment *in vivo* . Nat Commun. (2022), 1–14. doi: 10.1038/s41467-022-33787-8, PMID: 36245034 PMC9573882

[66] Boucher A Anderson C Hinman R Kindschuh M Fung J Wang T. Engineered human B cells targeting tumor-associated antigens exhibit antigen presentation and antibody-mediated functions. Front. Immunol. (2025) 16. 10.3389/fimmu.2025.1621222 PMC12343648 40808959

[67] Samorski R Gissmann L Osen W. Codon optimized expression of HPV 16 E6 renders target cells susceptible to E6-specific CTL recognition. Immunol Lett. (2006) 107:41–9. doi: 10.1016/j.imlet.2006.07.003, PMID: 16949679

[68] Lin KY Guarnieri FG Staveley-O’Carroll KF Levitsky HI August JT Pardoll DM. Treatment of established tumors with a novel vaccine that enhances major histocompatibility class II presentation of tumor antigen. Cancer Res. (1996) 56:21–6., PMID: 8548765

[69] Kürten CHL Kulkarni A Cillo AR Santos PM Roble AK Onkar S. Investigating immune and non-immune cell interactions in head and neck tumors by single-cell RNA sequencing. Nat Commun. (2021), 1–16. doi: 10.1038/s41467-021-27619-4, PMID: 34921143 PMC8683505

[70] van Haasteren J Li J Scheideler OJ Murthy N Schaffer DV. The delivery challenge: fulfilling the promise of therapeutic genome editing. Nat Biotechnol. (2020) 38:845–55. doi: 10.1038/s41587-020-0565-5, PMID: 32601435

[71] Elias A Kassis H Elkader SA Gritsenko N Nahmad A Shir H. HK022 bacteriophage Integrase mediated RMCE as a potential tool for human gene therapy. Nucleic Acids Res. (2020), 1–13. doi: 10.1093/nar/gkaa1140, PMID: 33270859 PMC7736782

[72] Barzel A Paulk NK Shi Y Huang Y Chu K Zhang F. Promoterless gene targeting without nucleases ameliorates haemophilia B in mice. Nature. (2015) 517:360–4. doi: 10.1038/nature13864, PMID: 25363772 PMC4297598

[73] Ashhurst TM Marsh-Wakefield F Haryono Putri G Spiteri AG Shinko D Read MN. Integration, exploration, and analysis of high-dimensional single-cell cytometry data using Spectre. Cytometry. (2021) 101(3):237–53. doi: 10.1101/2020.10.22.349563, PMID: 33840138

[74] Van Gassen S Callebaut B Van Helden MJ Lambrecht BN Demeester P Dhaene T. FlowSOM: Using self-organizing maps for visualization and interpretation of cytometry data. Cytometry. (2015) 87:636–45. doi: 10.1002/cyto.a.22625, PMID: 25573116

[75] McInnes L Healy J Melville J. UMAP: uniform manifold approximation and projection for dimension reduction. arxiv. (2020).

[76] Wickham H Averick M Bryan J Chang W McGowan L François R. Welcome to the tidyverse. J Open Source Software. (2019) 4:1686. doi: 10.21105/joss.01686

[77] Brinkman EK Chen T Amendola M Van Steensel B. Easy quantitative assessment of genome editing by sequence trace decomposition. Nucleic Acids Res. (2014) 42:1–8. doi: 10.1093/nar/gku936, PMID: 25300484 PMC4267669

