# Supplementary material for: Mouse B cells engineered to express an anti-HPV antibody elicit anti-tumor T cell responses
Source: Front Immunol. 2025 Jul 18;16:1613879. doi: 10.3389/fimmu.2025.1613879 (PMC12313569; doi:10.3389/fimmu.2025.1613879)
Supplement: Supplementary file 1 [file DataSheet1.pdf]

TITLE:

Mouse B cells engineered to express an anti-HPV antibody elicit anti-tumor T cell responses

AUTHORS:

Michal Guberman Bracha[1,2,†], Guy Biber[1,†], Natalie Zelikson[3], Sharon Shavit[1], Roy Avraham[1], Yaron Vagima[1,4], Débora Rosa Bublik[1], Yael Katz[1], Adi Barzel[2], Leah Natasha Klapper[1], Shmulik Hess[1], Alessio David Nahmad[1,5,]*

AFFILIATIONS:

[1] Tabby Therapeutics Ltd, Israel
[2] Faculty of Life Sciences, Tel Aviv University, Tel Aviv, Israel
[3] Faculty of Medical & Health Sciences, Tel Aviv University, Tel Aviv, Israel
[4] Department of Biotechnology, Israel Institute for Biological Research, Ness Ziona, Israel
[5] The Samueli Integrative Cancer Pioneering Institute, Israel

† These authors contributed equally: M. Guberman, G. Biber
** Corresponding author: alessionahmad@gmail.com*

SUPPLEMENTARY FIGURES:

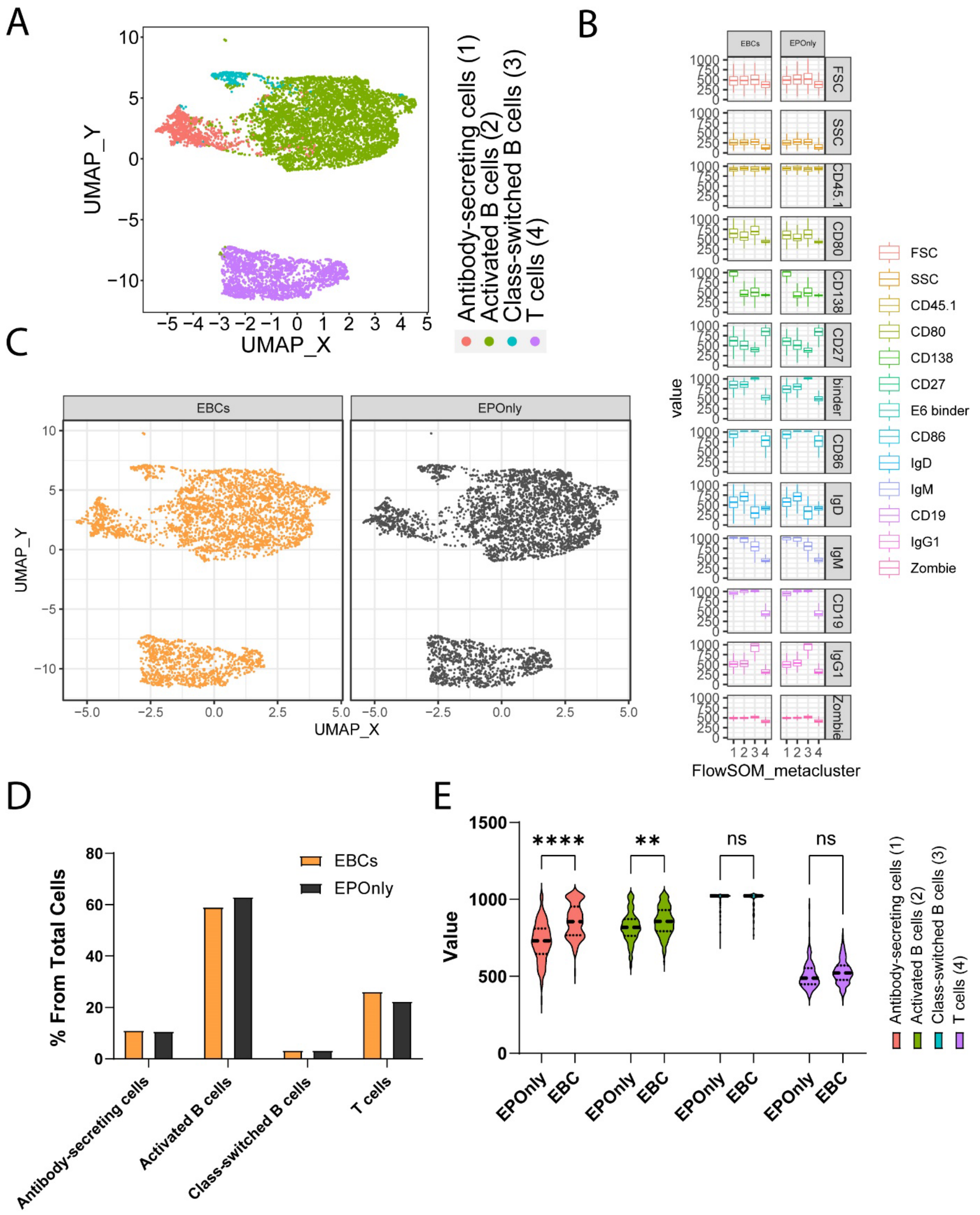

Supplementary Figure 1: EBC phenotype is similar to non-engineered B cells

A. UMAP plot of pooled spectral cytometry data of EPOnly and EBC cells. The resulting clusters were manually annotated based on marker expression as Antibody-secreting cells (1) Activated B cells (2) Class-switched B cells (3) and T cells (4). **B.** Breakdown of marker expression (rows) for either EBC or EPOnly cells (columns, left and right respectively) and cluster (marked on the x-axis in each column). CD45.1 was used as a pan-lymphocyte marker. **C.** Same as A. but this time cells from EBCs and EPOnly are separated. A similar clustering appears. **D.** Quantification of the frequencies of each cluster and comparison between EBCs and EPOnly control cells. **E.** Cluster-

wise analysis for E6 binding. Violin plots represent values for each cell as in A-B. Median and quartiles are indicated. ns=pv>0.05, **=pv<0.01, ****=pv<0.0001 for two-way ANOVA with Šidák's multiple comparisons tests. For A-E, representative experiment reproduced twice with independent binders.

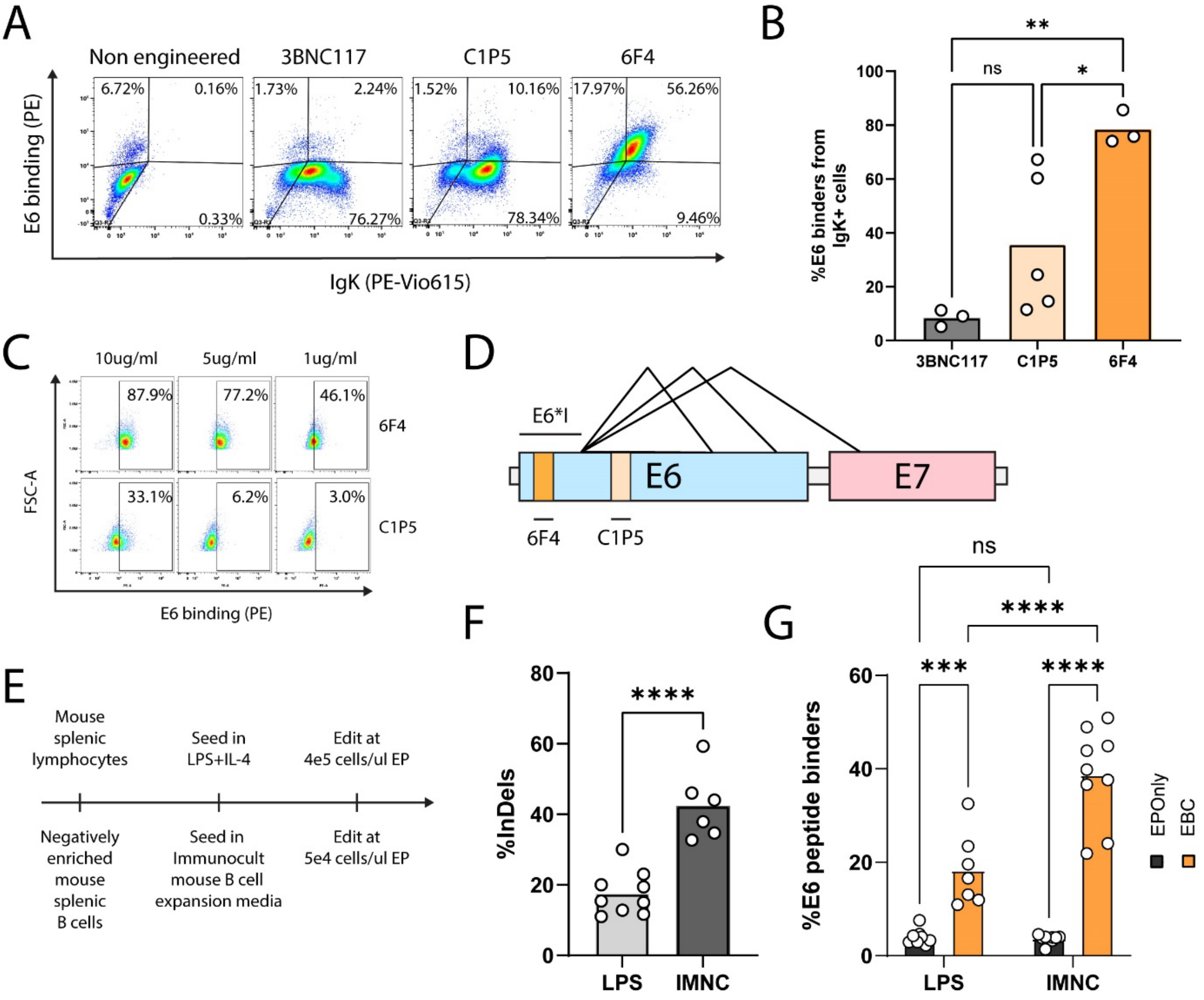

Supplementary Figure 2: Mouse B cell engineering optimizations

A. Flow cytometry example of i.29 cells engineered to express the anti-HIV-gp120 3BNC117 control antibody, anti-HPV-E6 C1P5 antibody or anti-HPV-E6 6F4 antibody. Engineered cells are detected by IgK expression. Pre-gated on live, singlet cells. **B.** Quantification of E6 binders from the IgK+ population of i.29 cells engineered to express either 3BNC117, C1P5 or 6F4, as in A. Each dot represents an independent flow cytometry experiment, n=3-5. ns=pv>0.05, *=pv<0.01, **=pv<0.001 for one-way ANOVA with Tukey's multiple comparison test. **C.** Flow cytometry example of i.29 cells engineered to express either 6F4 (top row) or C1P5 (bottom row) while utilizing 10ug/ml, 5ug/ml or 1ug/ml E6 concentrations in the staining procedure (columns, left to right respectively). **D.** Scheme of the E6 (blue) and E7 (red) genes in HPV. The 6F4 and C1P5 binding epitopes are indicated in orange. The E6*I, one of the functional transcripts enabling E7 expression, is indicated by a line above the represented gene. Splicing is represented by triangles above the genes. **E.** Representative comparison of the original (above) and optimized (below) mouse B cell engineering process. **F.** TIDE efficiency, as indicated by Insertions and deletions (InDels) in the original editing process (LPS) compared to the optimized engineering process (IMNC). Each dot represents an independent experiment, n=6-9. ****=pv<0.0001, two-tailed t-test. **G.** Engineering rates in the original editing process (LPS) compared to the optimized

engineering process (IMNC). Each dot represents an independent experiment, n=7-10. ns=pv>0.05, ***=pv<0.001, ****=pv<0.0001 for two-way ANOVA with uncorrected Fisher's LSD. For F,G the LPS data is also presented in Figure 2.

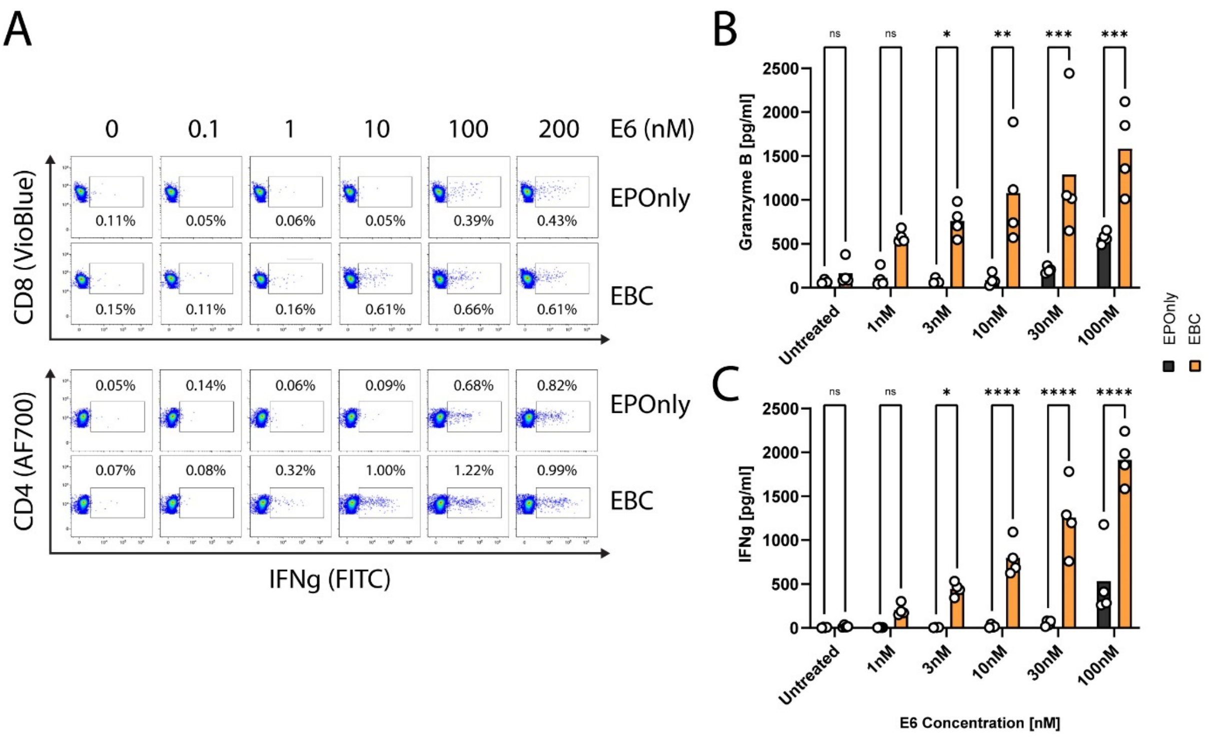

Supplementary Figure 3: EBCs activate polyclonal T cells in a dose dependent manner

A. Intracellular flow cytometry example of dose response curves of E6 loaded EBCs for the activation of CD8 (top) and CD4 (bottom) polyclonal T cells from immunized mice. EPOnly control B cells (top row for each panel) are compared to EBCs (bottom row for each panel) at varying E6 concentrations (columns) ranging from 0 to 200nM. Pre-gated on singlets, cells, alive, CD4- CD8+ (above) or CD4+ CD8- (below). **B.** ELISA for the secretion of Granzyme B (GrzmB) in the supernatant of EBC (orange) or EPOnly (black) cocultures with polyclonal anti-E6 T cells from immunized mice at varying concentrations of E6 pre-loaded to B cells. **C.** Same as B. but for IFNg secretion. For B-C, n=4 and ns=pv>0.05, *=pv<0.05, **=pv<0.01, ***=pv<0.001, ****=pv<0.0001 for two-way ANOVA with Šidák's multiple comparisons tests.
